# Single-incision laparoscopic ileocolectomy for solitary cecal colon diverticulitis with calcified fecalith: a case report

**DOI:** 10.1093/jscr/rjac323

**Published:** 2022-08-31

**Authors:** Akito Shimizu, Masanori Yoshimitsu, Takuya Yano, Ichiya Chogahara, Sotaro Fukuhara, Kanyu Nakano, Hitoshi Idani, Masazumi Okajima, Michihiro Ishida, Daisuke Satoh, Yasuhiro Choda, Yasuhiro Shirakawa, Hiroyoshi Matsukawa, Shigehiro Shiozaki

**Affiliations:** Department of Surgery, Hiroshima City Hiroshima Citizens Hospital, Hiroshima, 730-8518, Japan; Department of Surgery, Hiroshima City Hiroshima Citizens Hospital, Hiroshima, 730-8518, Japan; Department of Surgery, Hiroshima City Hiroshima Citizens Hospital, Hiroshima, 730-8518, Japan; Department of Surgery, Hiroshima City Hiroshima Citizens Hospital, Hiroshima, 730-8518, Japan; Department of Surgery, Hiroshima City Hiroshima Citizens Hospital, Hiroshima, 730-8518, Japan; Department of Surgery, Hiroshima City Hiroshima Citizens Hospital, Hiroshima, 730-8518, Japan; Department of Surgery, Hiroshima City Hiroshima Citizens Hospital, Hiroshima, 730-8518, Japan; Department of Surgery, Hiroshima City Hiroshima Citizens Hospital, Hiroshima, 730-8518, Japan; Department of Surgery, Hiroshima City Hiroshima Citizens Hospital, Hiroshima, 730-8518, Japan; Department of Surgery, Hiroshima City Hiroshima Citizens Hospital, Hiroshima, 730-8518, Japan; Department of Surgery, Hiroshima City Hiroshima Citizens Hospital, Hiroshima, 730-8518, Japan; Department of Surgery, Hiroshima City Hiroshima Citizens Hospital, Hiroshima, 730-8518, Japan; Department of Surgery, Hiroshima City Hiroshima Citizens Hospital, Hiroshima, 730-8518, Japan; Department of Surgery, Hiroshima City Hiroshima Citizens Hospital, Hiroshima, 730-8518, Japan

## Abstract

The prevalence of colonic diverticular disease has been on the increase in Japan due to an increase in westernized diet and a rapidly aging population. However, solitary cecal diverticulum is rare and considered congenital in etiology. Solitary cecal diverticulitis with calcified fecaliths is even rarer. Herein, we report a case of cecal colon diverticulitis caused by a calcified fecalith in a 38-year-old woman treated with single-incision laparoscopic surgery. To the best of our knowledge, this report describes the first case of cecal colon diverticulitis caused by a calcified fecalith that was successfully treated with single-incision laparoscopic ileocolectomy.

## INTRODUCTION

In Japan, opportunities to treat patients with colonic diverticular disease (diverticular bleeding and diverticulitis) have been increasing in recent years because the prevalence of colonic diverticula has increased [1] A solitary cecal diverticulum is considered to be congenital in etiology, and a true diverticulum is defined as a projection, including the mucosa, submucosa and muscularis propria [2]. To our knowledge, a few studies have shown that fecalith may result in diverticulitis and become calcified and visible [3]. Herein, we report a patient, in whom the fecalith became calcified and visible; therefore, we were able to make an appropriate preoperative diagnosis. Surgical management of nonperforated cecal diverticulitis remains controversial [4]. However, because of cosmesis, we chose ileocecal resection using single-port laparoscopic surgery (SILS) for the solitary cecal diverticulitis.

## CASE PRESENTATION

**Figure 1:**
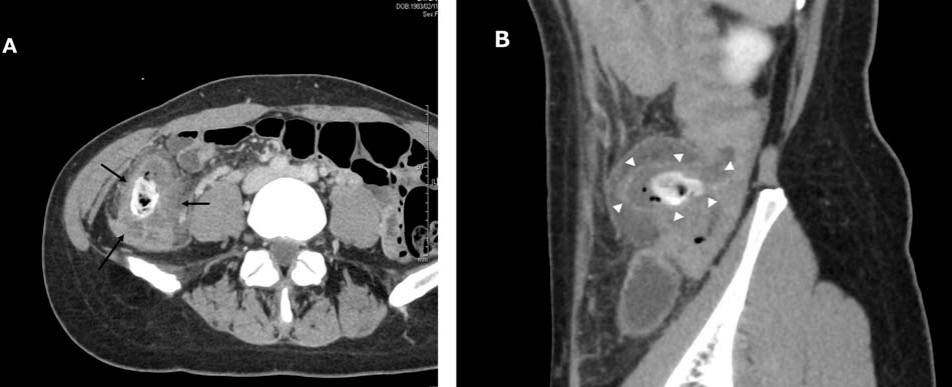
Abdominal and pelvic CT with intravenous contrast at the time of consultation. Axial image (**A**) demonstrated cecal colon diverticulum (black arrows) and sagittal image (**B**) demonstrated thickening and mural edema of the diverticulum (white arrowheads), indicating inflammation.

**Figure 2:**
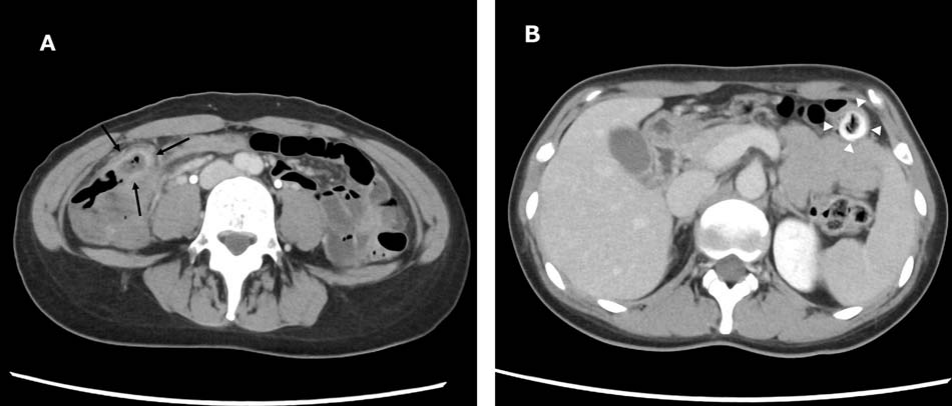
Abdominal and pelvic CT on hospitalization Day 3. Axial images (**A** and **B**) showing improved cecal colon diverticulitis (black arrows); however, fecaliths migrated to the transverse colon (white arrowheads).

A 38-year-old woman presented with lower right abdominal pain of 3 days duration before the consultation. She had a history of left ovarian cystectomy and no history of medication use or allergies. She was 172-cm tall and weighed 59.0 kg. On examination, there was right lower abdominal pain with no rebound tenderness. The white blood cell count was 13 600 /μl, and the C-reactive protein was 3.8 mg/dl. Contrast-enhanced computed tomography (CT) revealed a 4-cm cecal diverticulum with inflammation and a fecalith (Fig. 1). An abscess penetrated through the mesenteric side and was localized in the mesentery. The diagnosis was cecal colonic diverticulitis and classified as Hinchey II. The abdominal symptoms were localized; hence, the patient was treated with antibiotics. From Day 1 of hospitalization, she was on nil per oral and administered CMZ at 3 g/day. On hospitalization Day 3, the right lower abdominal pain improved, and contrast-enhanced CT imaging was performed. The cecal colonic diverticulitis improved, and the fecaliths migrated to the transverse colon (Fig. 2). Moreover, the inflammatory reaction improved. In a multidisciplinary meeting, we discussed that the patient should undergo a detailed examination, including colonoscopy, and elective surgery after readmission. However, we suggested that surgical treatment should be performed as early as possible after considering the risk of early recurrence and bleeding. The patient wished to undergo minimally invasive surgery; thus, we performed an immediate ileocolectomy and lymphadenectomy using SILS as we did not rule out the likelihood of malignant disease.

**Figure 3:**
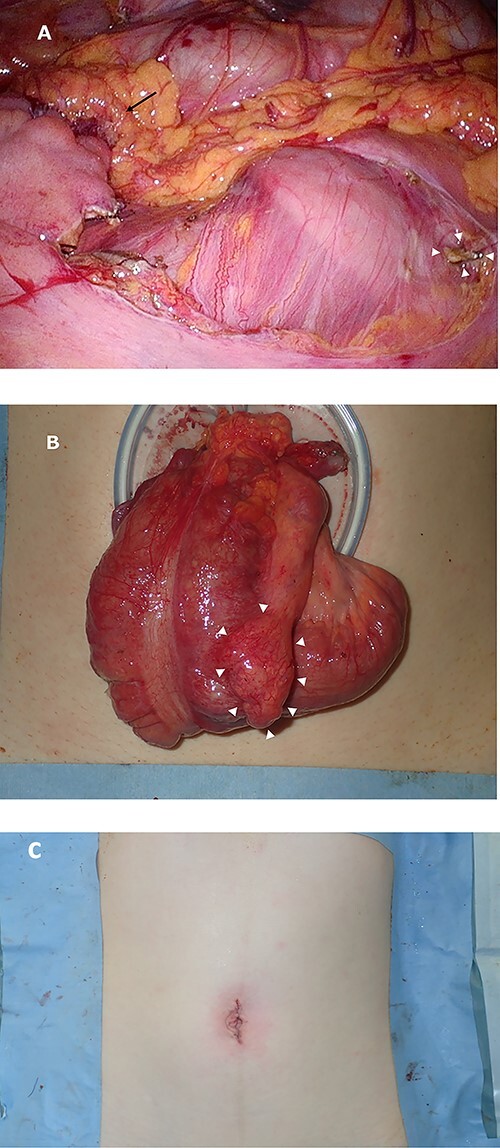
Intraoperative images. Image (**A**) demonstrating the anastomotic site (black arrows) and ileocolic artery and veinous dissection (white arrowheads). Image (**B**) showing the ileocecal region raised outside the body and a diverticulum (white arrowheads). Image (**C**) shows after closure.

**Figure 4:**
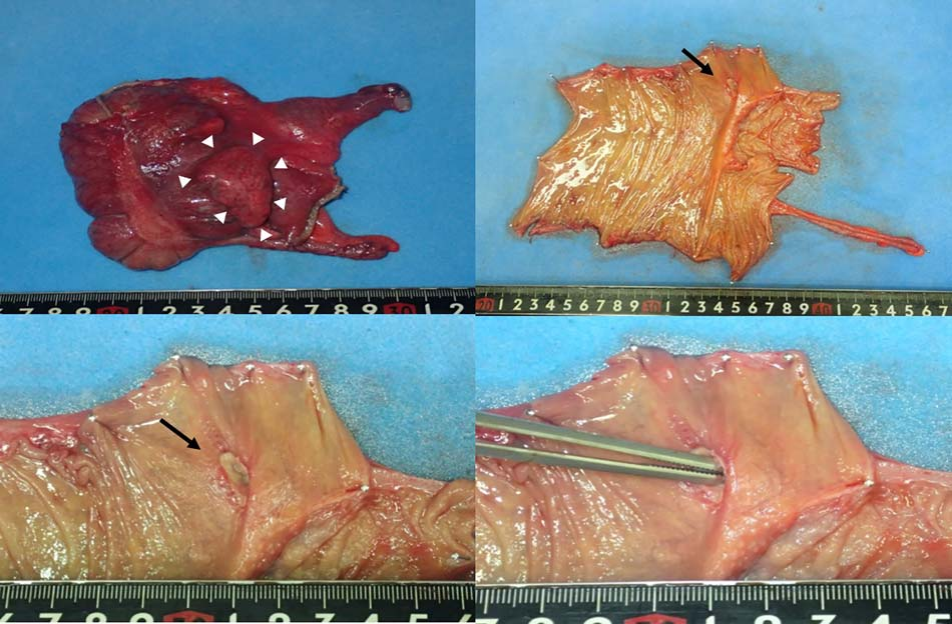
Macroscopic findings of the surgical specimen showing a diverticulum with an ulcer in the cecal colon (white arrowheads) and no fecalith (black arrows).

Laparoscopic findings showed that the inflammatory changes were not significant. The surgeon had expertise skills on multiport laparoscopic surgery (MLS) with some SILS experience. Initially, a 40-mm incision was made at the umbilicus. We then performed ileocolic mobilization and lymphadenectomy with ileocolic arterial and venous resection by SILS, similar to MLS (Fig. 3A). Functional end-to-end anastomosis was performed out of the body after removal through an umbilical incision (Fig. 3B and C). The operation time was 2 h and 47 min, and the amount of bleeding was minimal. Macroscopic findings of the surgical specimen showed a diverticulum with an ulcer in the cecal colon, but no fecalith (Fig. 4). Pathological findings showed deep ulceration and abscess formation with no malignant findings. The patient’s course was good, and she was discharged 8 days after the operation.

## DISCUSSION

The prevalence of colonic diverticular disease has been increasing in Japan due to the rapidly aging population. The Japanese Gastroenterological Association decided to create clinical guidelines for colonic diverticular bleeding and colonic diverticulitis [1]. There is no mention of the relationship between diverticulitis and fecalith in the guidelines. Few studies have shown that fecalith may result in diverticulitis and become calcified and visible [3]. The entrapped stool can transform into a fecalith of harder consistency, which can mechanically irritate the mucosal lining, resulting in a ‘stercoral trauma’ of the epithelial lining [5].

Solitary cecal diverticulitis is a rare cause of abdominal pain in Western patients and is more common in the Oriental population [6]. Solitary cecal diverticula are believed to be congenital in origin and arise as an outpouching of the cecum at 6 weeks of gestation [2]. Approximately 10% of diverticulitis are reported to be indistinguishable from carcinomas on CT [6]. Although the surgical management of nonperforated cecal diverticulitis is controversial, we decided to perform a minimally invasive and cosmetic ileocolectomy with lymphadenectomy. Considering the patient’s wish and oncological curability, she ultimately underwent ileocolectomy and lymphadenectomy using SILS. As a result, the lesion was diagnosed as benign; however, 14 lymph nodes were resected. Considering the number of resected lymph nodes, surgery was an acceptable procedure even if the lesion was malignant.

Single-incision laparoscopic surgery is a less invasive surgery than MLS; however, it is known to be a difficult maneuver [7]. During the surgery, we did not observe any adhesions or inflammatory reactions around the cecum. Considering the natural course of inflammation in diverticulitis, the lesion may have been in the early phase. In addition, the patient was lean with no history of major abdominal surgery, which allowed her to successfully undergo a minimally invasive, cosmetically beneficial and oncologically acceptable procedure without any particular difficulties during the surgery. SILS has recently been used for the resection of benign and malignant gastrointestinal tumors [8, 9]. To our knowledge, this is the first report of SILS for solitary cecal diverticulitis. We believe that SILS can be performed safely for solitary cecal diverticulitis, when inflammation is mild in adults and should be considered if contraindications are not present.

## AUTHORS’ CONTRIBUTIONS

MY contributed to the conceptualization, data curation, writing of the original draft and editing. TY, SF, IC, KN, HI and MO contributed to the supervision, writing of the original draft and editing.

## REGISTRATION OF RESEARCH STUDIES

This paper is a clinical report; therefore, the authors declare that no registration was required.
